# Chicken Is a Useful Model to Investigate the Role of Adipokines in Metabolic and Reproductive Diseases

**DOI:** 10.1155/2018/4579734

**Published:** 2018-06-19

**Authors:** Namya Mellouk, Christelle Ramé, Alix Barbe, Jérémy Grandhaye, Pascal Froment, Joëlle Dupont

**Affiliations:** INRA, UMR 85 Physiologie de la Reproduction et des Comportements, 37380 Nouzilly, France

## Abstract

Reproduction is a complex and essential physiological process required by all species to produce a new generation. This process involves strict hormonal regulation, depending on a connection between the hypothalamus-pituitary-gonadal axis and peripheral organs. Metabolic homeostasis influences the reproductive functions, and its alteration leads to disturbances in the reproductive functions of humans as well as animals. For a long time, adipose tissue has been recognised as an endocrine organ but its ability to secrete and release hormones called adipokines is now emerging. Adipokines have been found to play a major role in the regulation of metabolic and reproductive processes at both central and peripheral levels. Leptin was initially the first adipokine that has been described to be the most involved in the metabolism/reproduction interrelation in mammals. In avian species, the role of leptin is still under debate. Recently, three novel adipokines have been discovered: adiponectin (ADIPOQ, ACRP30), visfatin (NAMPT, PBEF), and chemerin (RARRES2, TIG2). However, their mode of action between mammalian and nonmammalian species is different due to the different reproductive and metabolic systems. Herein, we will provide an overview of the structure and function related to metabolic and reproductive mechanisms of the latter three adipokines with emphasis on avian species.

## 1. Introduction

Adipose tissue was initially recognised only as an energy storage organ. Since the discovery of leptin, an increasing number of studies have reported that adipose tissue may also play a role as a dynamic endocrine organ by synthesising and secreting numerous bioactive factors termed adipokines [1–4]. In mammals, these molecules are involved in the regulation of multiple biological processes such as metabolism (glucose and fatty acid) and reproduction (steroidogenesis, gonadal development, and gametogenesis).

Energy homeostasis is mostly dependent on lifestyle, including physical activity and a healthy diet (food variety and intake), and also on hormonal regulation and a genetic predisposition to metabolic diseases like obesity. Globally, the number of obesity cases has almost tripled since 1975 and has become a major public human health problem. In 2016, the number of overweight (body mass index (BMI) ≥ 25 kg/m^2^) or obese (BMI ≥ 30 kg/m^2^) people reached 1.9 billion people in the world (World Health Organisation 2016). People that suffer from this pathology have a high risk of developing type 2 diabetes, insulin resistance, cardiovascular disease, and infertility [5]. One of the female reproductive pathologies that may be associated with obesity and insulin resistance is polycystic ovary syndrome (PCOS). PCOS is characterised by the consensus of Rotterdam as a syndrome of ovarian dysfunction presenting 2 of the following 3 criteria: oligo- or anovulation, clinical and/or biochemical signs of hyperandrogenism, and polycystic ovaries without any sign of other aetiologies (congenital adrenal hyperplasia, androgen-secreting tumours, and Cushing's syndrome) [6]. Approximately 75% of PCOS patients are overweight and central obesity is observed in both normal and overweight PCOS women [7, 8]. One of the potential biochemical tools that can be used to give an overview of the state of reproductive health is the measurement of serum adipokines. Indeed, adipokines have recently been shown to be increased in the serum of overweight/obese PCOS patients compared to normal-weight patients [9–14]. In males, obesity has been linked to hypogonadism as well as to a reduction of sperm quantity and quality [15, 16]. These impairments appear when the endocrine system is altered. In fact, in obese men, there is excessive activity of cytochrome P450-alpha, leading to an increase in the conversion of androgen into oestrogen and a decrease of testosterone levels [17]. Consequently, testosterone and FSH plasma levels are negatively correlated with BMI [18] and testosterone levels increase after bariatric surgery [19]. In addition, rising plasma levels of leptin and chemerin are observed, while those of adiponectin are decreased in obese men [20–22]. The relationship with other adipokines is still obscure, even though some studies have focused on their molecular role [23]. Metabolic diseases also affect farm animals, especially chickens, because of the genetic and nutritional practices used to optimise meat and for egg production. The domestic chicken represents both a widely used biomedical model and an important source of high-quality protein in the human diet. Despite decades of intensive genetic selection, the remarkable growth rate of commercial broiler chickens is still improving but is also accompanied by deleterious increases in body fat and skeletal muscle and disorders in metabolism and reproduction.

In this review, we report several traits that make chicken a viable model for studies of adipose biology, obesity, and insulin resistance. Most metabolic genes are conserved in humans, and a number of quantitative trait loci (QTLs) that have been linked to fatness in chickens contain genes implicated in human susceptibility to obesity or diabetes [24]. In addition, a recent study described the differential expression of adipokines in adipose tissue of two lines of meat-type chickens that have been genetically selected for either high (FL) or low (LL) visceral abdominal fatness [25]. In addition, overfeeding of hens led to reproductive deficiencies linked to the anarchic follicular hierarchy for females and a delay in sexual maturation in males [26, 27]. Finally, the egg presents an opportunity to directly manipulate the developmental milieu and study the consequences on adipose metabolism via in ovo injection. These peculiarities make chickens a good animal model to understand the relationship between adipokines, metabolism, and reproduction and their associated mechanisms.

The most studied adipokine in mammals was leptin, but its existence in avian species faced extensive controversies for a long time. Nowadays, the long list of adipokines reached more than a hundred and included adiponectin, visfatin, and chemerin, which control glycaemia, energy, and fertility homeostasis [23, 28]. Their structures and physiological functions were largely described in mammals, particularly in humans and rodents, but less is known about their involvement in avian species. Furthermore, several adipokines found in mammals like TNF*α*, resistin, and omentin have not been mapped to the chicken genome [29]. Recently, the chicken genes of three novel adipokines (adiponectin, visfatin, and chemerin) were cloned and evidence showed their potential role as key regulators of food intake, muscle growth, and reproduction [30–32] in avian species; however, knowledge of their functional activity needs to be expanded.

In the current review, after a brief description of the metabolic and reproductive peculiarities of avian species and the impact of metabolism on reproduction in this species, we will focus on the structure and function of three adipokines (adiponectin, visfatin, and chemerin) with regard to chicken metabolism and reproduction.

## 2. Metabolic Peculiarities in Avian Species

The metabolic system of chicken is closely related to that in mammals. Glucose is stored as glycogen in tissues and used for energy production through glycolysis. Glucose is the exclusive source of energy for the brain. However, chickens constitutively exhibit “hyperglycaemia” (>200 mg/dL), despite rather normal levels of a hyperactive endogenous insulin. Large doses of exogenous insulin are required to induce hypoglycaemia; furthermore, chickens tolerate doses of exogenous insulin that would be lethal to mammals [33, 34]. The release of insulin by the perfused chicken pancreas also appears unusual in response to metabolites, which are insulinotropic in nondiabetic mammals [35, 36]. Therefore, chickens are constitutively hyperglycaemic and insulin resistant, which makes chickens mimic the condition of type 2 diabetes in mammals [37]. Glycaemia levels depend on the line, age, and sex of the animals [38], and enhanced adiposity in chickens is associated with lower fasting plasma glucose, which is in contrast to the situation in mammals [39]. This has been observed in four experimental chicken lines that were genetically selected for fatness (FL) versus leanness (LL) [40] or high growth (HG) versus low growth (LG) [41], where the HG chickens are also fatter than LG chickens. Interestingly, FL chickens are clearly not hyperphagic, since they eat the same amount of feed as LL chickens. Furthermore, FL chickens are not resistant to exogenous insulin; in fact, the FL birds are more sensitive to the hypoglycaemic effect of insulin than LL chickens [42]. Reciprocally, divergent selection for high or low fasting plasma glucose levels induces an associated change in adiposity, where chickens with low fasting plasma glucose are also fatter [39, 43].

In mammals, insulin sensitivity of the various tissues is an important factor controlling nutrient partitioning. Any alteration of the insulin signalling cascade in one of the major metabolic tissues (liver, muscle, or fat tissues) will alter nutrient utilisation and storage and ultimately body composition. The peculiarities exhibited by chickens for plasma glucose levels and insulin action compared with mammals have been described in different reviews [34, 37]. Insulin exerts pleiotropic effects in chicken [44]. To date, insulin receptors, two receptor substrates (IRS-1 and Shc), and major downstream components of insulin signalling have been characterised in chicken liver, muscle, and adipose tissue in different experimental models [45–47]. Insulin signalling appears to proceed through tissue-specific cascades in chicken metabolic tissues. In the liver, insulin elicits a signalling cascade with a similar response to those observed in mammals, including tyrosine phosphorylation of the insulin receptor *β*-subunit (IR*β*), insulin receptor substrate-1 (IRS-1), and Src homology 2 domain-containing substrate (Shc) and activation of phosphatidylinositol 3-kinase (PI3K) [45, 46]. The situation in skeletal muscle is very different. Tyrosine phosphorylation of IR*β* and IRS-1 and PI3K activity are not regulated by insulin, whereas event downstream of PI3K (e.g., Akt and P70S6K activation) is accordingly sensitive [46]. Furthermore, in several skeletal muscles, chickens and ducks are not totally insensitive to exogenous insulin, which enhances the uptake of glucose [48, 49]. Moreover, immunoneutralisation of insulin rapidly induces considerable increases in plasma levels of glucose in young chickens [44]. Insulin induces a rapid although modest increase in glucose uptake by chicken myotubes, an uptake that is inhibited by phloretin, an inhibitor of glucose transporters [50]. These findings support the existence of functional glucose transporters in avian muscle. Nevertheless, the mechanism of the control of plasma glucose in chickens remains to be elucidated as immunoreactive GLUT1, but no GLUT4 has been detected in chicken tissues. Recently, Coudert et al. suggested that the facilitative glucose transporter protein GLUT12 could act in chicken muscle as an insulin-sensitive transporter that is qualitatively similar to GLUT4 in mammals [51, 52]. In chicken adipose tissue, as in muscle, we reported that insulin also does not elicit a classical IR*β*-initiated cascade, including the downstream steps of Akt and P70S6K activation [47].

The chicken metabolic system was submitted to large changes since their body weight and fat are approximately four times heavier than 50 years ago [53]. The increase in adipose tissue mass was needed to assume the huge requirement of meat and egg production [54]. The abdominal (visceral) fat pad is the major fat tissue in chickens. Adipose tissue growth is a combination of hyperplasia during young age and hypertrophia in adult chickens mostly, which contributes to fat deposition [55]. It expands rapidly during post hatch. Chicken adipocytes increase volume by storing fatty acids that come primarily from the liver. In both chickens and humans, the liver serves as the primary site of de novo lipogenesis, whereas the rate of lipogenesis in adipose tissue is about 100 times lower. Hormonal and nutritional control of hepatic lipogenesis is comparable between birds and mammals. In chicken, lipogenesis is low in adipose tissue as compared to liver. Furthermore, the regulatory mechanisms of lipid metabolism can be different in these two tissues. As previously described, the existence of the insulin-dependent glucose transporter (GLUT4) has not been established in chickens. No direct effect of insulin on glucose transport has been shown in chicken adipocytes, although an increase in glucose disappearance from the incubation medium of cultured chicken adipocytes has been taken as indirect evidence of an effect of insulin on glucose transport [56]. In isolated chicken adipose tissue or adipocytes, insulin slightly stimulates glucose oxidation and the incorporation of acetate-U-^14^C into lipids in the presence of glucose. Compared to rat adipocytes, the insulin stimulation of lipogenesis is slow (~3 hours), is low in magnitude (30–40%), and requires very high insulin concentrations [36].

## 3. Reproductive Peculiarities in Chicken Species

In all birds, the female is the heterogametic sex (ZW), while the male is homogametic (ZZ). In contrast to mammals, female chickens maintain only the left reproductive tract (ovary plus oviduct). The ovary is typically organised in a strict follicular hierarchy consisting of 2 to approximately 6 preovulatory follicles and ovulates at most a single follicle per day. Physiologically, only the largest preovulatory follicle ovulates every 26–28 h. The characteristics of ovarian asymmetry and preovulatory follicle hierarchy are generally believed to be at least in part reflections of weight reduction for flight [57]. In ad libitum (free access to food) fed hens, the ovarian follicular hierarchy is disorganised by multiple ovulations resulting in fertility deficiency and ovarian cancer [58, 59]. As in mammals, steroidogenesis in preovulatory follicles occurs within multiple layers of the theca. In birds, theca cells express aromatase and synthesise oestrogens from androgen precursors that are localised to the externa while pregnenolone, progesterone, and androgen precursors are produced almost exclusively within the theca interna [60]. The granulosa cells produce progesterone, de novo, from cholesterol and pregnenolone and has the capacity to convert progesterone to testosterone but not to oestrogen. In contrast to mammals, ovulation in birds is induced by the stimulatory action of ovarian progesterone derived predominantly from the granulosa layer of the largest preovulatory follicle and pituitary LH.

Interestingly, the ovary of the aging domestic hen has been utilised as a model for human reproductive cancers. This is based upon observations that the hen develops spontaneous ovarian/oviductal tumours with high incidence (estimated in 30–35% of hens by 3.5 years of age); the tumours are associated with the accumulation of ascites fluid; plus, they biochemically and histologically resemble human tumours of epithelial origin [61]. Furthermore, birds offer excellent models to study the mechanism and function of hormone-mediated maternal effects since the embryo develops outside the mother's body, facilitating the measurement and manipulation of early hormone exposure. Finally, another peculiarity in female birds is that the oviduct is able to store sperm for a prolonged period. The sperm storage tubules (SST) are located in the uterovaginal junction of the oviduct, where sperm can be stored and survive for a few weeks after insemination or natural mating [62]. Nowadays, the advantage of prolonged sperm storage and survival in the oviduct of laying hens is utilised in practical poultry production systems. Indeed, this peculiarity enables laying hens to produce a series of fertile eggs following a single copulation event or artificial insemination. In the male chicken prepubertal layer, anaerobic glycolysis in the testis may participate in sertoli cell proliferation, which may improve meiotic processes and consequently sperm production [63]. Finally, unlike mammals, birds do not possess a pampiniform complex (venous and arterial complex which makes it possible to maintain the intratesticular temperature constant).

## 4. The Metabolic Impact on Reproductive Performances in Chicken Species

In birds, like all other species, nutrition, more particularly energy metabolism, influences the reproductive function. Models of hyperphagic birds have shown the negative effect of overfeeding in both sexes, while a dietary restriction during their growth increases oviposition rates and the duration of the fertile period.

### 4.1. Relationship between Growth, Restriction, and Fertility

In chicken selected for meat production, the rapid growth of selected individuals is almost always accompanied by an impairment of maximum reproductive capacity in both sexes [64]. The case of broiler lines is typical: the selection of fast-growing lines for more than 60 years was accompanied, in the males of these lines, by a very high sexual precocity. This great “spontaneous” precocity results in the appearance of testicular spermatozoa from the age of 11-12 weeks in roosters. It also results in relatively low maximum testicular development and testicular regression from the age of 43–45 weeks. In addition, an increasing proportion of these cocks (around 40–60% of the total) showed a shorter breeding season, which leads to replacing them well before the end of the laying period in females, sometimes causing severe problems in social behaviour. In 1990, Reddy and Sadjadi estimated that males had a decreased ability to fertilise eggs by about 0.5% for each new generation [65]. However, the excessive growth of males is usually accompanied by hyperphagic behaviour due to the overconsumption of food in relation to their needs. In females, the increase in weight induces the anarchic development of follicles, which can lead to the coexistence of several follicular hierarchies that disrupt ovulation [66]. A relationship between the weight of chickens at sexual maturity and the number of large follicles growing on the ovary has been shown [67].

For both sexes, the maintenance of reproductive performance (e.g., spawning and fertility) according to the standard of the strain can be assured only if strict food restriction is applied at a very young age (2-3 sem after hatching). Thus, the control of body weight via food restriction makes it possible to preserve in males (a) a morphology and reduction of locomotor disorders induced by overweightness, compatible with mating [68]) and (b) an acceptable fertility at least during the first part of the sexual season [69–71]. However, it appears that the application of restrictions will have side effects on behaviour, such as pecking [72].

### 4.2. Overfeeding-Fertility Relationship

In birds, as previously described, liver function has some specificities compared to mammals. During food intake, the lipids absorbed in the intestine will first cross the liver, where they can be collected and used before reaching the bloodstream [73]. In addition, the intake of dietary carbohydrates will stimulate lipid synthesis. In birds, the liver is also the main site of de novo lipogenesis, including triglyceride synthesis and also, as in mammals, phospholipids and cholesterol [74]. These triglycerides produced by the liver are either incorporated in VLDL (very low-density lipoprotein) and then transported by the blood to growing oocytes, adipocytes, and muscle tissue or can be stored in the liver. In birds, the liver also produces vitellogenin, which acts on ovarian function. The ovary can also interact with liver activity, since both vitellogenin and hepatic VLDL production are stimulated by oestrogen [75].

Although there is no real obesity in poultry, unlike mammals, the overweightness observed is rather associated with fast-growing lines (strain meat) that can have a behaviour of hyperphagia. Models of overweight birds (ad libitum food or gavage) have provided a better understanding of the consequences of overfeeding on reproductive function in both sexes. Thus, in roosters, gavage-induced obesity decreases sperm production by 50% and reduces egg production in chicken. In males, this drop in fertility is the result of a decrease in testicular weight (approximately 30% in 4 weeks), which is accompanied by a decrease in testosterone and an increase in intratesticular cholesterol and an internal temperature of 0.3°C [76]. Thus, the increase in temperature following the increase in energy resulting from gavage would lead to an alteration in the functional state of the spermatogonia stem, thereby causing a decrease in the production of spermatozoa.

In the immature female, significant body weight is often a consequence of excessive consumption in comparison to their needs, which causes the accelerated development of the reproductive system at the time of sexual maturity [77, 78] and ovarian hyperactivity. Although the production of an egg is a process requiring a lot of energy, an excess of energy decreases the production of functional oocytes by causing dysregulation of the follicular hierarchy [79]. An increase in the frequency of multiple ovulations or close ovulation leads to a higher incidence of abnormal eggs (deformed, soft, etc.). Multiple ovulations lead to the appearance of “double eggs” (eggs with two egg yolks), whereas close ovulations usually lead to the appearance of a first “normal” egg, with the second being smaller, deformed, and presenting calcification defects [80]. Walzem et al. studied the effect of overfeeding on hepatic lipoprotein production using the laying hen as a model. They observed an increase in the diameter of lipid vesicles of VLDL type, which have the peculiarity in hens of having an identical size of approximately 30 nm. This alteration in physicochemical properties modifies the blood transport to the follicle under development [80]. Eventually, this lack of transport leads to a cessation of yolk deposition in growing follicles. In such females, moderate quantitative restriction or a limitation of dietary energy intake (“qualitative” restriction) is usually sufficient to restore the optimal ovulation rate [78, 81, 82].

All together, these findings show that chickens, in the same way as mammals, do not escape reproductive disorders in the case of metabolic dysfunction. Various hormones, including growth hormone, insulin-like growth factors (IGFs), and insulin, have been proposed as potential mediators affecting reproductive function. However, the interactions between the reproductive endocrine axis and the metabolic axis have not been clearly determined. Adipokines represent good candidates for such reproductive-metabolic interactions.

## 5. Leptin Controversy in Avian Species

In mammals, leptin was discovered as the first obesogenic gene in 1994 by Zhang et al. [4]. Clinical investigations as well as mice in vivo studies proved that leptin is a key regulator of energy homeostasis and mediates satiety signals to the central nervous system [83]. Leptin secreted from adipocytes is clearly positively related to adipose tissue masses and is secreted more by subcutaneous than visceral adipose tissue [84]. In accordance with this finding, circulating leptin remains elevated in obese patients and is associated with reproductive functions [85, 86]. The leptin gene has also been cloned in other mammalian species such as primates, rodents, and porcine, ovine, bovine, and canine species and shares a close homology with the mouse leptin gene [87–92]. However, in chicken, subsequent studies have brought conflicting results regarding leptin gene cloning. First, Taouis et al. and Ashwell et al. reported avian leptin sequences, after which Friedman-Einat et al. contradicted their findings [93–95]. Thus, the leptin gene was considered for a decade to be missing from the avian genome. However, 2 or 3 years ago, leptin genes were discovered in several bird species: in zebra finch (*Taeniopygia guttata*), rock dove (*Columba livia*), falcon (*Falco peregrinus*), and quail (*Coturnix japonica*) [96–99]. More than 20 years after the characterisation of leptin in mammals, Seroussi et al. identified the leptin (LEP) genes of chicken (*Gallus gallus*) and duck (*Anas platyrhynchos*) [100, 101]. These newly identified avian LEP proteins share only 26–30% identity with human LEP. This group suggests an autocrine/paracrine mode of action for bird leptin instead of it being a circulating hormone, as in mammals. Chicken leptin mRNA was highly correlated with leptin receptor (LEPR) expression (except in the pituitary) and was reported to be mostly expressed in the brain, with LEPR expressed mostly in the pituitary. Similar to other avian species and conversely to humans, chicken leptin mRNA is not highly expressed in adipose tissue and, similar to zebra finch, is not expressed in the liver [97, 100]. The intramuscular administration of leptin antibodies induces feed intake and increases glycaemia and lipaemia, which mimic the effect of leptin depletion in the ob/ob mouse model, and increases the expression of the leptin receptor in adipose tissue, the liver, and muscle [102]. Moreover, a recent study showed no effect of a chicken leptin peptide on food intake or behaviour, suggesting that chicken leptin is not sufficient to mediate effects on appetite in the brain [103]. The in vivo injection of leptin also improved the negative effects of fasting on ovarian function by attenuating follicular apoptosis, delaying the cessation of egg laying and influencing ovarian steroidogenesis [104]. Thus, the role of leptin in avian species is still unclear and chicken leptin likely has a different physiological role in birds than in mammals. Two independent studies report that approximately 274 to 640 protein-encoding genes that are present in the genomes of most vertebrate lineages including humans are missing from 60 bird genomes [105, 106]. A recent study based on the phylogenic evolution of genome supported the hypothesis that other adipokines, including TNF*α*, resistin, and omentin, might be missing from the chicken genome [29]. However, Lovell et al. brought new arguments to contradict the absence of some genes in the bird genome, especially due to their location in GC-rich regions and the technical limitations to identifying them [107]. Based on this hypothesis, Bornelöv et al. conducted a de novo transcriptome assembly and identified 191 new GC-rich genes in chickens, including TNF*α* [108]. One year later, Rohde et al. reported the identification and functional characterisation of the avian orthologue of TNF*α* [109]. An additional study also indicated that TNF*α* mRNA was poorly expressed in the visceral fat of female broilers and layer chickens and was not affected by feed deprivation [110]. These recent data open new debates on the inexistence of other adipokines considered missing from the chicken genome; if they are identified, more investigations will be needed to determine their potential involvement in the endocrine control of metabolic and reproductive functions in chicken.

## 6. Adiponectin

### 6.1. Structure and Expression of Adiponectin and Its Receptors

Adiponectin cDNA was isolated from human adipose tissue in 1996 by Maeda et al. as the adipose most abundant gene transcript 1 (apM1) [111] and in parallel from murine fibroblast cell lines (AdipoQ) by Hu et al. [112]. The 15.8 kb adiponectin gene encodes a 26 kDa protein that was described for the first time by Scherer et al. and designated as adipocyte complement-related protein (ACRP30) [113]. The adiponectin protein was also extracted from human plasma [114], where it was considered the most abundant adipokine, ranging between 5 and 30 mg/L. Adiponectin is secreted into the blood from adipocytes with a higher serum level associated with the female gender and inversely related to body weight. It is found in cells and plasma in three major forms: trimers, hexamers, and high-molecular weight (HMW) [115]. In addition, a smaller fragment generated by the proteolytic cleavage of full-length adiponectin gives rise to a globular domain of protein gAd which is secreted in the plasma. Among them, the HMW form plays important roles in the regulation of insulin signalling and is closely associated with peripheral insulin sensitivity [116]. In patients with obesity or type 2 diabetes, plasma levels of HMW adiponectin are decreased [117, 118] and a reduction in HMW adiponectin levels, rather than total adiponectin levels, contributes to the aetiology of obesity-associated diseases [119]. Adiponectin is able to bind three kinds of receptors: AdipoR1, AdipoR2, and T-cadherin. The first two consist of seven transmembrane domains, with the opposite topology to G-protein-coupled receptors in which the N-terminal region is cytoplasmic, while the C-terminal region is extracellular [120]. The binding of adiponectin to AdipoR1 preferentially results in the activation of AMPK pathways, whereas the adiponectin/AdipoR2 interaction induces the stimulation of the PPAR*α* (peroxisome proliferator-activated receptor alpha) signal. These receptors, although expressed ubiquitously, have different tissue distributions. AdipoR1 has a predominant location in skeletal muscle and endothelial cells, while AdipoR2 is mainly expressed in the liver. The third receptor is a glycosyl-phosphatidylinositol receptor, belonging to the cadherin family, which lacks a transmembrane domain. The intracellular signalling connected to this receptor seems to require other unidentified coreceptors or AdipoR1/AdipoR2.

In chicken, the coding region of chicken adiponectin shares 67% and 65% identity with human and mouse, respectively [121]. In addition, the chicken ADIPOR1 cDNA was found to be 80–83% homologous to human, mouse, rat, or pig ADIPOR1 cDNA, while the deduced protein sequence was 91% similar to mammalian ADIPOR1. Similarly, the chicken ADIPOR2 cDNA was 76–78% homologous to human, mouse, or pig ADIPOR2 cDNA, while the deduced protein sequence was 82% similar to mammalian ADIPOR2 [122]. Adiponectin and adiponectin receptor genes are ubiquitously expressed in various tissues (Table 1) [123, 124], and the expression of the adiponectin system (adiponectin, ADIPOR1, and ADIPOR2) in adipose tissue and muscle depends on the gender and age of the animals [125]. In adipose tissue, adiponectin mRNA was higher in 154-day-old females than in males and ADIPOR1 mRNA was higher in 154-day-old males than in females. Adiponectin and ADIPOR2 mRNA were higher, and ADIPOR1 mRNA was lower, in thigh muscle in female compared with male chickens. Furthermore, the adiponectin plasma levels are lower in 8-week-old chickens which have more abdominal fat pad mass relative to body weight than 4-week-old chickens, suggesting that adiposity or age influence the adiponectin plasma levels in chickens [126]. In addition, the adiponectin gene may be associated with the initiation and growth processes of adipose tissue deposition in chickens [127, 128]. Chicken fed ad libitum develop more abdominal adipose tissue which is accompanied by an increase in adiponectin mRNA expression in adipose tissue [128].

### 6.2. Role of Adiponectin

In mammals, basic science studies have shown the beneficial effects of adiponectin on various physiological functions, including glucose homeostasis, food intake, apoptosis, oxidative stress, and atherosclerotic processes; so, this molecule usually has been considered a beneficial adipokine [129, 130]. For example, adiponectin is known to play key roles as an insulin sensitiser and an anti-inflammatory regulator, in addition to the regulation of glucose metabolism and fatty acid breakdown [130]. In wild-type and diabetic mice, a two- to five-fold increase in circulating adiponectin levels can reduce plasma glucose levels [131]. The injection of adiponectin in obese and type 1-diabetes mice models displaying hyperglycaemia and severe hyperinsulinaemia restored normal circulating levels of glucose [131]. Furthermore, adiponectin knockout mice fed with a high-fat diet develop glucose intolerance and severe hepatic insulin resistance [132]. Chronic treatment with globular adiponectin resulted in decreased body weight and adipocyte areas in high-fat diet-fed rats accompanied by an increase in PPAR*γ* expression in adipose tissue that prevents the dysregulation of lipolysis [133]. There were strong inverse associations between circulating HMW adiponectin and intramyocellular lipid content in human skeletal muscle [134]. Adiponectin also enhances AMPK activity in the arcuate hypothalamus (ARH) via its receptor AdipoR1 to stimulate food intake in mice [135]. In addition, several reports have indicated an association between low adiponectin levels and an elevated risk of various cancers (breast, endometrial, and gastric). Concerning the reproductive functions, plasma adiponectin levels were found to be 4-fold higher in sexually mature versus sexually immature mice [136]. In the ovary, adiponectin and its receptors appear to be involved in steroidogenesis in a different manner depending on the species [137]. In human granulosa cells, both FSH and hCG (as a surrogate for LH) treatment increased AdipoR2 mRNA by more than 2-fold and stimulation with adiponectin improved hCG-induced progesterone production 3-fold [138]. In porcine follicular cells, adiponectin increases steroidogenic acute regulatory protein (StAR) transcript abundance but reduces cytochrome P450 aromatase expression [139]. Similarly, adiponectin inhibits insulin-induced progesterone and androstenedione production in bovine theca cells [140]. In our lab, we showed that adiponectin decreases insulin-induced steroidogenesis and increases IGF1-induced proliferation of cultured bovine granulosa cells [141]. In males, the expression of AdipoR2 appears to be critical for testicular function since AdipoR2-deficient knockout mice exhibit reduced testis weight characterised by atrophy of the seminiferous tubules and aspermia, while plasma testosterone levels remained unaffected [142]. Stimulation with recombinant adiponectin also inhibited basal and human hCG-stimulated testosterone secretion in rat-cultured Leydig cells [143]. The role of adiponectin in the hypothalamic-pituitary-gonadal axis and in the PCOS pathology has been recently reported by Rak et al. [137].

In broiler chickens, Tahmoorespur et al. showed that adiponectin mRNA expression in adipose tissue was inversely related to chicken belly fat deposition levels [128]. Adiponectin has a remarkable effect on the impairment of adipocyte differentiation, which contributes to the negative regulation of fat deposition in chicken [144]. Yan et al. observed that adiponectin inhibited lipid deposition and the differentiation of chicken preadipocytes through the p38 MAPK/ATF-2 and TOR/p70 S6 kinase signalling pathways [145]. Chicken globular adiponectin inhibits lipid deposition in adipocytes by suppressing the expression of CEBP and FAS, while increasing the expression of ATGL. The mechanism is explained by the observations that globular adiponectin stimulates p38 MAPK/ATF-2 activation and suppresses the TOR/p70 S6 kinase pathway [146]. More precisely, the ADIPOR1 gene is implicated in metabolism and/or fat deposition in broilers [147]. In chicken adipocytes, adiponectin also regulates mitochondrial biogenesis by inhibiting lipid accumulation and activating the AMPK/ACC signalling pathway [148]. In the muscle of broilers, rosiglitazone (antidiabetic drug) increases circulating adiponectin levels while dexamethasone (glucocorticoid anti-inflammatory drugs) has opposite effects and adiponectin has an antilipogenic effect through the p38 MAPK/ATF2 signalling pathway [145]. For the same body weight and egg production, the high residual feed intake chicken line (R^+^: fat line) consumes 40% more food than their counterpart low residual feed intake chicken line (R^−^: lean line). In the hypothalamus, ADIPOR1 expression is increased in R^+^ as compared to R^−^ chickens, suggesting a role for this receptor in food intake regulation in chicken [149]. In reproduction, the adiponectin gene was found in the chicken ovary to be mainly expressed in theca cells and is suggested to exert a paracrine or autocrine effect on ovarian steroidogenesis. Adiponectin increased IGF-1-induced progesterone secretion in F2 and F3/4 follicles, whereas it halved progesterone production in response to LH and FSH in F3/4 follicles [150]. In male broiler breeder chickens, the expression of adiponectin and its receptors has been studied in testes [151]. A significant elevation of ADIPO1 and ADIPOR2 gene expression is observed in sexually mature chickens, which could be a result of the higher metabolic activity related to spermatogenesis, testicular steroid hormone production, and the transportation of spermatozoa and testicular fluid [151].

**Figure 1:**
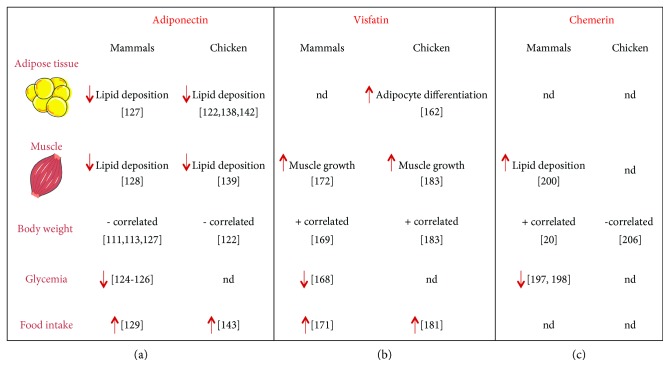
Comparison of adiponectin (a), visfatin (b), and chemerin (c) effects on main metabolic functions in mammal versus chicken. nd: not determined; + correlated: positively correlated; − correlated: negatively correlated; ↑: increase; ↓: decrease.

**Figure 2:**
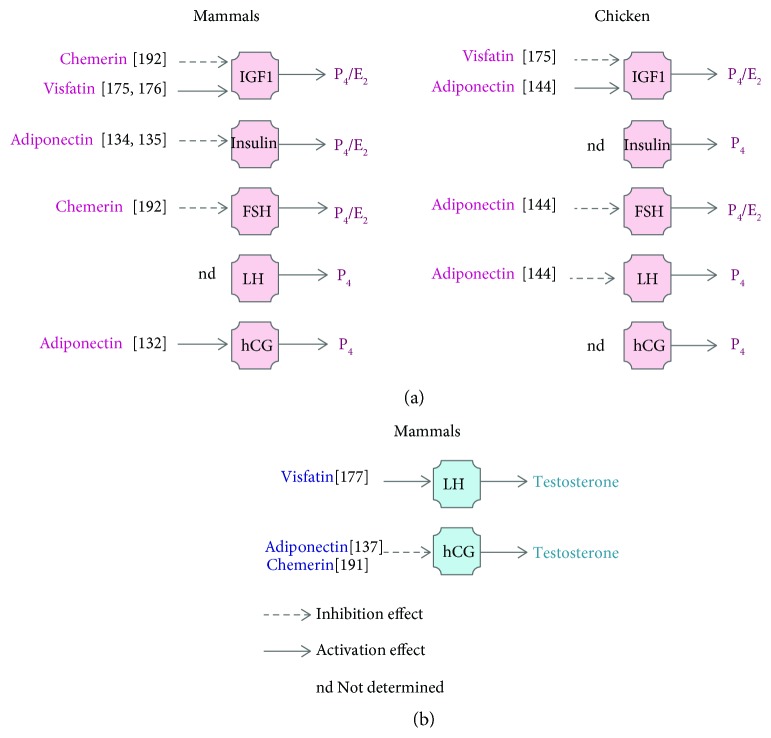
Comparison of adiponectin, visfatin, and chemerin effects on female (a) and male (b) steroidogenesis in mammal (a, b) versus chicken (a). IGF1: insulin like growth factor 1, FSH: follicle-stimulating hormone, LH: luteinizing hormone, hCG: human chorionic gonadotropin.

Globally, adiponectin limits lipid deposition in adipose tissue and induces food intake through AdipoR1/AMPK signalling in the human and chicken adipose hypothalamus (Figure 1(a)). Also, adiponectin as an insulin sensitiser could be tested in chicken, especially for their natural insulin resistance. However, the effects of adiponectin on steroidogenesis are dependent on the species, suggesting different physiological regulations (Figure 2).

## 7. Visfatin

### 7.1. Structure and Expression of Visfatin

Visfatin was first discovered as a growth factor called pre-B cell colony-enhancing factor (PBEF) in 1994 from human peripheral blood lymphocytes that are able to initiate the maturation of B-cell precursors (135). Visfatin has also been considered a type II nicotinamide phosphoribosyltransferase (NAMPT) due to its ability to synthesise nicotinamide mononucleotide (NMN) from nicotinamide and 5′-phosphoribosyl-1′-pyrophosphate. NMN is a therapeutic target for treating metabolic disorders by improving glucose clearance in obese and diabetic mice models [152–154]. The identification of visfatin as an adipokine has been controversial since an active binding site of the insulin receptor was discovered. The adipogenic and insulin mimetic action of visfatin depends on the preparation of recombinant visfatin. To date, only four different recombinant visfatin forms were validated [155–157]. Recently, the crystal structure of rat [158, 159], mouse [160], and human [161, 162] visfatin has been solved and revealed a dimer organisation separated by an active site. In humans, the visfatin gene is on the long arm of chromosome 7 and encodes a 52 kDa secreted protein [163]. Visfatin expression has been studied to a large extent in humans and also in animal models [164–166] including chicken [167].

The full length of the chicken visfatin gene has been cloned from adult liver. The chicken visfatin protein had high amino acid sequence similarities with those of humans (94%), rodents (94%) [167] and other agronomic species (94%) [168]. The chicken visfatin mRNA was detected in many tissues such as the brain, heart, intestine, kidney, liver, lung, muscle, spleen and gonads (Table 1) [167, 169]. Not surprisingly, visfatin was also expressed in adipose tissue without any difference between subcutaneous and visceral fat tissues in humans [170] and chicken [167]. However, Li et al. showed that visfatin was differentially expressed in adipose tissue depending on the chicken species, with higher mRNA levels in broiler chicken (fast growing) than in silky flow (low growing), suggesting a potential role as a marker of fat accumulation [168]. In addition, visfatin expression is sexually dimorphic and depends on tissue types. In chicken, it was described more as a myokine than an adipokine, because of its main expression in muscle and its ability to decrease the expression of MYF5 expression (a myogenic factor) in myoblasts [31]. One of our recent studies also demonstrated that visfatin was more expressed in the theca than in granulosa cells in turkeys [32] and that its plasma level was higher at the end of the laying period compared to the beginning. We also described its expression in the ovarian cortex, granulosa, and theca cells of chicken hierarchical follicles. To date, no visfatin receptor has been identified.

### 7.2. Role of Visfatin

Visfatin is a pleiotropic protein involved in a large spectrum of physiological processes from aging to atherosclerosis [171]; here, however, we will focus only on metabolic and reproductive functions. Physiological studies have revealed a strong role of visfatin on glucose, fatty acid metabolism, and muscle growth. A loss of visfatin in mice adipose tissue impaired adipose tissue functions such as inflammation, severe insulin resistance via the synthesis of nicotinamide that is one of the oldest drugs known for its antilipolytic effects [172], mediated by its interaction with GPR109A, a receptor on the adipocyte plasma membrane [173]. Visfatin also improves glucose-stimulated insulin secretion in pancreatic *β*-cells by increasing nicotinamide adenine dinucleotide biosynthesis, while visfatin haplodeficiency causes impaired glucose tolerance in mice, which was rescued after NMN administration [174]. Visfatin has become an emerging adipokine due to subsequent studies that have brought proof regarding it positive association with obesity and type 2 diabetes [175, 176]. In rats, the injection of visfatin in the arcuate nucleus of the hypothalamus plays an orexigenic role via the modulation of dopamine, CART, and CRH peptide activity [177]. In addition, the depletion of visfatin in mice leads to a decrease in intramuscular NAD synthesis and consequently induced fibre degeneration and progressive loss of strength and treadmill endurance muscle [178]. On the other hand, the visfatin concentration profile in follicular fluid is a potential indicator for ovarian reserve for woman undergoing ovarian stimulation regarding to the positive correlation between its expression in follicular fluid and the number of oocyte retrieved [179]. The visfatin expression in ovarian mice increased with advancing follicular development [180]. Choi et al. [180] also reported that the administration of low concentrations of visfatin during superovulation improved the fertility of aged female mice. Furthermore, visfatin increases IGF-1-induced progesterone and oestradiol production in human and bovine-cultured granulosa cells [181, 182]. Visfatin protein expression was detected in human sertoli cells and Leydig cells and in the tail and the connecting piece of spermatozoa. Visfatin protein expression and release are higher in immature than in mature ejaculated spermatozoa leading to the increased production of nicotinamide without any effect on sperm mobility and viability [183]. Visfatin also seems to be involved in the regulation of rat testicular activity since its testicular decreasing expression is positively correlated with serum testosterone levels and testis weight in a diabetic rat model [184]. In addition, visfatin increases testicular steroidogenesis from purified rat Leydig cells [185].

In chickens, there is increasing evidence that visfatin is involved in the regulation of muscle growth [186], metabolism [31], food intake, and reproductive functions [187, 188]. For instance, a polymorphism in exon 7 of the visfatin gene was positively associated with the body weight of 4- and 6-week-old chickens, as well as the body slanting length, fat bandwidth, breast muscle water loss rate, and breast muscle fibre density and breastbone length of 4-week-old chickens [189]. The central injection of visfatin in chicks induced an increase in their food intake, suggesting that visfatin is a potent orexigenic factor [187]. In addition, supplementing the chicken diet with chenodeoxycholic acid induced a decrease in feed intake and body weight associated with a reduction of the expression of visfatin in the liver. This suggests a potential role of visfatin in hepatic lipogenesis [190]. Similarly to insulin, recombinant chicken visfatin may induce the differentiation of 3T3-L1 cell lines by increasing the mRNA expression of adipocyte differentiation markers (PPAR*γ*, aP2, FAS, and C/EBP*α*) [168]. Visfatin also acts in reproductive tissues such as ovarian (theca and granulosa cells) and testicular cells (sertoli cells, Leydig cells, and spermatozoa). More precisely, visfatin inhibits IGF1-induced progesterone production in hen granulosa cells and its protein levels in the testis and plasma increase in adults compared to prepubertal chickens, suggesting a potential role in regulating testosterone production [188, 191].

Thus, chicken visfatin and mammalian visfatin act as an orexigenic factor, regulating muscle growth, and their expression is positively correlated with body weight (Figure 1(b)). However, chicken visfatin plays an opposite role on male and female steroidogenesis compared to mammals (Figure 2). This makes chicken a good model to deepen our knowledge on the regulatory mechanisms induced by visfatin in food intake, adipogenesis, and myogenesis. On the other hand, the insulin mimetic activity of visfatin could be interesting to confirm in chicken.

## 8. Chemerin

### 8.1. Structure and Expression of Chemerin

**Figure 3:**
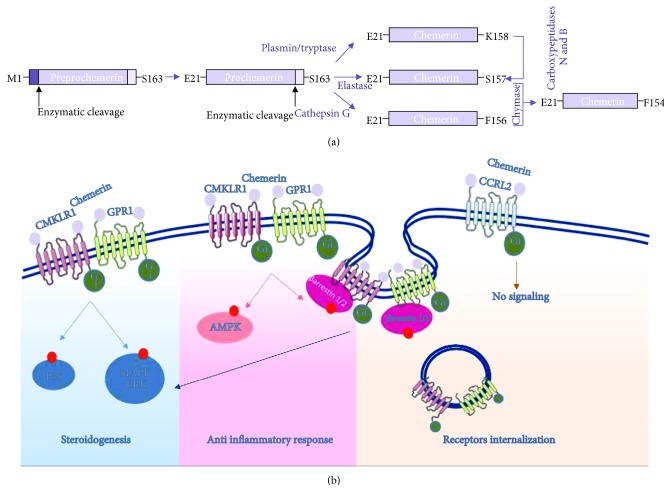
Structure of chemerin (a) and representation of chemerin system signalling (b). CMKLR1 (chemokine-like receptor); GPR1 (G protein-coupled receptor 1); and CCRL2 (chemokine (C-C motif) receptor-like 2).

Chemerin is an adipose cytokine which was previously known as tazarotene-induced gene 2 (TIG2) and retinoic acid receptor responder protein 2 (RARRES2) [192]. Chemerin is a recently identified adipokine that is closely related to the pathogenesis of metabolic syndrome [193]. It is secreted as a 143-amino acid inactive prochemerin, which is then hydrolysed by the enzymatic cleavage of 5 to 7 amino acids from its carboxyl terminus in the extracellular compartment. Two neutrophil serine proteases, elastase and cathepsin G, remove 6 and 7 amino acids, respectively, to generate an active form. Plasmin and tryptase are also able to cleave 5 amino acids from the carboxyl terminus followed by cleavage of the carboxyl-terminal lysine by carboxypeptidases N and B that also result in the active chemerin [194] (Figure 3(a)). Chemerin is secreted from white adipocytes and expressed in several tissues, mainly white and brown adipose tissue and the liver, pancreas, placenta, skin, kidney, adrenal gland, lung, intestine, ovary, and testis [195–198]. Chemerin exerts its physiological functions through the binding of three G protein-coupled receptors: the chemokine-like receptor 1 (CMKLR1), G protein-coupled receptor 1 (GPR1), and chemokine (C-C motif) receptor-like 2 (CCRL2) [195, 199]. CMKLR1 is coupled to the Gi/o family of G proteins and inhibits the cAMP signalling pathway while promoting phospholipase C, PI3K, MAPK, calcium mobilisation [194], and *β*-arrestin recruitment, which activate MAPK (ERK1/2) [200]. The GPR1 sequence is closely related to CMKLR1 with more than 40% identity and activates the same pathway [201]. In contrast, CCRL2 does not seem to promote any signalling pathway and does not induce receptor internalisation [200] (Figure 3(b)).

In avian species, chemerin and their receptors are expressed in peripheral tissues and ovarian cells (Table 1). In turkeys, chemerin mRNA was mainly present in the liver compared to the heart, adipose tissue, and muscles, while CMKLR1 and GPR1 mRNAs were ubiquitous. CCRL2 mRNA was highly expressed in pectoral muscle and adipose tissue compared to the liver, heart, and leg muscle. In addition, chemerin and its receptors were more expressed in theca cells compared to granulosa cells in both preovulatory follicle 1 and 3/4 hierarchical follicles [32].

### 8.2. Role of Chemerin

Chemerin is involved in the regulation of blood pressure, inflammation, immune responses, adipocytes differentiation, and carbohydrate metabolism and plays a key role in metabolic diseases, such as obesity and diabetes [202]. GPR1 knockout mice fed with a high-fat diet developed serious glucose intolerance and a test of pyruvic acid tolerance suppressed glucose-stimulated insulin levels that consequently increased glycaemia [203]. Similar results were observed in CMKLR1-knockout mice which developed exacerbated glucose tolerance and insulin sensitivity with no effects on high-fat diet-induced glucose intolerance after cold exposure [204]. In overweight/obese patients, the chemerin concentration is rising and is positively correlated with BMI and waist circumference [20]. Chemerin levels are reduced after bariatric surgery [205]. In bovine intramuscular adipocytes, chemerin also promotes lipolysis in mature adipocytes and adipogenesis during adipocyte differentiation [206]. These results suggest that the chemerin system could act on glucose and fat metabolism linked to obesity. Subsequent studies have described chemerin to inhibit gonad steroidogenesis from the testis and ovary and be involved in follicular development [196, 207]. We showed that chemerin decreased IGF- or FSH-induced progesterone and oestradiol secretion in cultured granulosa cells [198]. In addition, the level of chemerin is increased in the plasma and adipose tissue of patients with PCOS [208], as well as in the plasma of patients affected by preeclampsia [209]. Chemerin expression is also increased in the ovaries of rats treated with 5alpha-dehydrotestosterone (DHT) (mimicking PCOS) that is associated with a decrease in oestradiol secretion in granulosa cells and induces apoptosis [210]. In mice with CMKLR1 gene deletions, the effects of chronic DHT treatment on ovarian function in experimental PCOS are largely reduced, suggesting a role of the chemerin system in PCOS pathology [211]. As already mentioned, PCOS syndrome is, in some cases, associated with insulin resistance, which can both be treated with antidiabetic drugs. Metformin, an antidiabetic agent, restores physiological plasma chemerin concentrations (around 2 ng/mL) and decreases chemerin protein expression in the adipose tissue of women with PCOS, while insulin increases them, confirming the interrelation between chemerin, insulin, and reproductive homeostasis [208]. Chemerin also exerts an important role in male reproductive functions, including gametogenesis and steroidogenesis. In humans, chemerin levels in seminal plasma are negatively correlated with sperm quantity, maturation, and motility [20]. Chemerin also inhibits in vitro hCG-induced testosterone secretion in primary cultured Leydig cells [197]. These findings suggest that chemerin can regulate steroid secretion in reproductive organs and may act as a key regulator of metabolic diseases such as obesity and PCOS.

Recently, we described that chemerin and its receptors are expressed in chicken adipose tissue, liver, muscle, and ovarian cells. Our results indicated that plasma chemerin levels are negatively correlated with the fattening state of broiler hens. We also found that a restricted diet applied from 3 to 39 weeks begins to increase the plasma chemerin levels in hens during the laying period (18–39 weeks) and decreases the mRNA expression of chemerin in the liver and adipose tissue compared to ad libitum hens at 39 weeks. Furthermore, fish oil supplied (1% of the diet) from 9 to 39 weeks decreased the plasma levels of chemerin from the beginning of the treatment to the end of the prepubertal period (21 weeks) in broiler hens and decreased the mRNA expression of CCRL2 in adipose tissue and muscle and those of CMKLR1 only in adipose tissue [212]. In addition, we found that chemerin was negatively correlated with the percentage of hatchability of fertile eggs in broiler hens and the weight of preovulatory follicle 1 was positively correlated with the expression of chemerin in granulosa cells and that the production of progesterone by granulosa cells was negatively correlated with the expression of chemerin in theca cells. Restrictedly fed hens expressed lower chemerin mRNA levels in theca cells from preovulatory follicles 1 and 3 than ad libitum-fed hens. Fish oil supplement (1% of the diet) increased the mRNA expression of CMKLR1 in theca cells of preovulatory follicle F1 and decreases those of chemerin in theca cells of preovulatory follicle F3 [213]. The chicken chemerin gene sequence shares 81% identity with the turkey chemerin sequence. In turkeys, the plasma concentration of chemerin decreases at the end of the laying period and is negatively correlated with levels of plasma cholesterol, triglycerides, and phospholipid levels during the entire laying period [32]. The literature on chicken chemerin is poorly enriched and further experiments are needed to understand its promising role in metabolism and reproduction.

Finally, not enough studies were conducted in chicken to draw conclusions. However, regarding our discoveries, chemerin seems to be involved in the regulation of chicken metabolism and reproduction but in an opposite way as in those of mammals (Figure 1(c)).

## 9. Conclusions

Reproductive dysfunction arising from metabolic dysregulation is mostly associated with obesity and other metabolic and reproductive syndromes in humans and farm animals. In this review, we reported that many researches have linked food intake, body and fat weight, and reproductive function to plasma adipokines levels or tissue expression, especially those of leptin, visfatin, and chemerin levels. We particularly focused on chickens that were submitted to various nutritional, metabolic, and reproductive changes due to their rapid growth and high production. Chicken is an atypical species in view of their natural hyperglycaemia, insulin resistance, hepatic fatty acid synthesis, and reproductive system. Studies on chicken adipokines are emerging, and, regarding physiological features, chicken appears as an interesting model for in vivo studies that may provide critical information on the roles of adipokines on lipid and carbohydrate metabolism and the link to reproductive physiology.

## Figures and Tables

**Table 1:** Expression of adipokines (visfatin, adiponectin, and chemerin) and adipokine receptors (ADIPOR1, ADIPOR2, CMKLR1, GPR1, and CCRL2) in the main metabolic and reproductive tissues in chicken.

		Adipose tissue	Liver	Muscle	Brain	Ovary	Testis	References
Visfatin	mRNA	+	+	+	+	+	+	[161, 162, 206, 207]
	Protein	nd	nd	+	nd	+	+	[180,185]

Adiponectin	mRNA	+	+	+	+	+	+	[119, 144, 145, 206, 207]
	Protein	+	+	+	nd	+	+	[117, 118, 121, 145]

Chemerin	mRNA	+	+	+	nd	+	nd	[206, 207]
	Protein	nd	nd	nd	nd	nd	nd	

ADIPOR1	mRNA	+	+	+	+	+	+	[119, 143, 144, 145, 206, 207]
	Protein	nd	nd	nd	nd	+	+	[118, 145]

ADIPOR2	mRNA	+	+	+	+	+	+	[119, 144, 145, 206, 207]
	Protein	nd	nd	nd	nd	+	+	[118, 145]

CMKLR1	mRNA	+	+	+		+		[206, 207]
	Protein	nd	nd	nd	nd	nd	nd	

GPR1	mRNA	+	+	+		+	nd	[206, 207]
	Protein	nd	nd	nd	nd	nd	nd	

CCRL2	mRNA	+	+	+		+	nd	[206, 207]
	Protein	nd	nd	nd	nd	nd	nd	

+: detected; nd: not determined.
